# An Exploratory Study of Predictors of Response to Vagus Nerve Stimulation Paired with Upper-Limb Rehabilitation After Ischemic Stroke

**DOI:** 10.1038/s41598-019-52092-x

**Published:** 2019-11-04

**Authors:** David Alexander Dickie, Teresa Jacobson Kimberley, David Pierce, Navzer Engineer, W. Brent Tarver, Jesse Dawson

**Affiliations:** 1 Institute of Cardiovascular and Medical Sciences, College of Medical, Veterinary & Life Sciences, University of Glasgow, Queen Elizabeth University Hospital, Glasgow, G51 4TF United Kingdom; 2 School of Health and Rehabilitation Sciences, MGH Institute of Health Professions, Charlestown Navy Yard, 36 1st Avenue, Boston, MA 02129 United States; 3 MicroTransponder, Inc., Austin, TX United States

**Keywords:** Translational research, Stroke

## Abstract

We have previously shown the safety and feasibility of vagus nerve stimulation (VNS) paired with upper-limb rehabilitation after ischemic stroke. In this exploratory study, we assessed whether clinical and brain MRI variables predict response to treatment. We used data from two completed randomised and blinded clinical trials (N = 35). All participants had moderate to severe upper-limb weakness and were randomised to 6-weeks intensive physiotherapy with or without VNS. Participants had 3 T brain MRI at baseline. The primary outcome was change in Fugl-Meyer Assessment, upper-extremity score (FMA-UE) from baseline to the first day after therapy completion. We used general linear regression to identify clinical and brain MRI predictors of change in FMA-UE. VNS-treated participants had greater improvement in FMA-UE at day-1 post therapy than controls (8.63 ± 5.02 versus 3.79 ± 5.04 points, *t* = 2.83, Cohen’s d = 0.96, *P* = 0.008). Higher cerebrospinal fluid volume was associated with less improvement in FMA-UE in the control but not VNS group. This was also true for white matter hyperintensity volume but not after removal of an outlying participant from the control group. Responders in the VNS group had more severe arm impairment at baseline than responders to control. A phase III trial is now underway to formally determine whether VNS improves outcomes and will explore whether these differ in people with more severe baseline upper-limb disability and cerebrovascular disease.

## Introduction

Vagus nerve stimulation (VNS) paired with upper-limb rehabilitation is a potential novel treatment for arm weakness after stroke. VNS triggers release of plasticity promoting neuromodulators, such as acetylcholine and norepinephrine, throughout the cortex^1^. Timing this with motor training drives task-specific plasticity in the motor cortex^2^ and VNS paired with rehabilitative training has been shown to improve recovery in different preclinical models of stroke, both in comparison to VNS alone and rehabilitation alone^3,4^. These improvements were associated with synaptic reorganization of cortical motor networks and recruitment of residual motor neurons controlling the impaired forelimb^5^. Two clinical studies comparing VNS paired with upper-limb rehabilitation with upper-limb rehabilitation alone have shown it to be acceptably safe and feasible and that it may improve arm weakness after ischemic stroke^6,7^.

Arm weakness is the most common symptom of stroke and approximately half of stroke survivors with arm weakness have prolonged disability, which is associated with reduced quality of life^8,9^. Restoration of arm function after stroke is a priority for many stroke survivors^10^. However, recovery of motor function after stroke varies, so identifying factors that help predict response is important to aid patient selection and identify those most likely to respond. This is particularly true where therapies are invasive (involving surgery) and/or time consuming; VNS requires implantation of a nerve stimulator which is costly and associated with risks of anaesthesia, a small risk of infection and small risk of vocal cord palsy. There are several clinical and brain imaging markers that predict cognitive and functional recovery after stroke including age, level of impairment, white matter hyperintensity (WMH) volume, stroke lesion volume, corticospinal tract damage and blood pressure level^11–13^.

In the present study, we combined clinical and brain magnetic resonance imaging (MRI) data from our two previous randomised trials of VNS paired with rehabilitation for the upper-limb after ischemic stroke^6,7^. We performed exploratory analyses to assess predictors of response to VNS paired with upper-limb rehabilitation. Our goal was to identify predictive factors for further study that may help with patient selection for this promising novel therapy.

## Methods

### Study design, participants, and therapy

We have previously performed two randomised, blinded clinical trials of VNS therapy paired with rehabilitation for upper-limb recovery after stroke (NCT01669161 and NCT02243020)^6,7^. Data from these two trials were combined in the present analysis. The first trial was a Prospective Randomized Open, Blinded End-point (PROBE) study. The second was fully-blinded with sham stimulation. Approval was granted by the Medicine and Healthcare Regulatory Authority and by the West of Scotland Research Ethics Committee. All procedures performed in human participants were in accordance with the ethical standards of institutional committees and with the 1964 Helsinki declaration and its later amendments or comparable ethical standards. Informed consent was obtained from all individual participants included in the study.

The trials have been described in detail elsewhere^6,7^. The first trial was a PROBE design with an active rehabilitation control and the second was a fully blinded study with sham stimulation where all participants were implanted with a VNS device. In both studies, the VNS device implantation was performed under general anaesthesia by a surgeon. The device (Vivistim® System, MicroTransponder Inc., Austin, TX) consisted of an implantable pulse generator (IPG, Model 1001, MicroTransponder Inc., Austin, TX), an implantable lead (2.0 mm or 3.0 mm, Model 3000, MicroTransponder Inc., Austin, TX), and a wireless transmitter (Model 2000, MicroTransponder Inc., Austin, TX).

Participants had moderate to severe upper-limb weakness defined as a score of 20–50 on the Fugl-Myer Assessment, upper-extremity (FMA-UE) in both trials. The FMA-UE score assesses movement quality and synergy and is a well validated assessment of impairment. It ranges from 0 to 66 points. An improvement of greater than 6 points is typically defined as a clinically significant change being associated with improvement in function and quality of life^14^. Other inclusion and exclusion criteria were similar across trials and were fully described previously^6,7^.

The therapy protocol for both active VNS and control groups included 6-weeks of intensive upper-limb rehabilitation. Participants did in-clinic rehabilitation three times a week for 6 weeks, for a total of 18 sessions. All sessions started with 10 to 15 minutes of stretching exercises, followed by seven standardized tasks. Each session lasted approximately 2 hours. Functional tasks included reach and grasp, opening doors and locks, gross movement, object flipping, simulated eating tasks, inserting objects, and opening containers. These standardized tasks were performed in this order during each therapy session. Participants did approximately 30–50 repetitions per task and spent 10–20 minutes doing each task. For each participant, tasks were graded in difficulty and adapted to each person’s abilities and goals. Therefore, the exact number of repetitions per task varied depending on the level of impairment.

The VNS group received active VNS combined with rehabilitation exercises, while the control group did the rehabilitation exercises without VNS pairing. The VNS stimulation settings in the active VNS group were 0.5 s of VNS (0.8 mA, constant current, charge balanced pulses, 100 µs pulse width, 30 Hz frequency) during each repetition of a therapy task in both trials. VNS was delivered by a push button, held in the therapist’s hand and connected wirelessly via a laptop to the VNS device, during each repetition in a therapy session. The mean number of stimulations per session were 444 for the VNS group and 511 for the control group. The control group had an outlier for number of stimulations per session and without their data the control group was similar to the VNS group at 464 stimulations per session. The mean number of stimulations per minute during each task was 4.9 and 5.6 for VNS and control groups, respectively.

In the first PROBE design study, the therapist used the push button in the control group during therapy sessions, but this was not connected to a VNS device as there was no sham implantation. In the second fully blinded study the push button connected wirelessly to the implanted VNS device in control participants but a 0.0 mA sham stimulation was delivered during each task.

Participants had baseline FMA-UE scores recorded and FMA-UE score was reassessed at the end of the 6 weeks in clinic therapy. After this 6-week visit, the therapy protocol differed between the two trials, but these data are not included in this combined analysis.

### Brain MRI acquisition and analysis

All participants had a structural brain MRI scan performed before randomisation unless contraindicated; the MRI protocol has been described in detail previously^15^. In brief, T1, T2, fluid attenuated inversion recovery (FLAIR), susceptibility-weighted (SWI), and diffusion tensor images were acquired using 3 T MRI. Stroke infarct volumes were manually segmented by an experienced image analyst (DAD) trained in accordance with STRIVE guidelines^16^. Segmentation of WMH and normal appearing tissue volumes was described previously^15^.

The corticospinal tract in each subject was estimated by diffeomorphically registering^17^ the “JHU DTI-based white-matter atlas”^18^ to each subject. Estimates of corticospinal tracts in each subject were visually inspected and approved. Whole brain mean diffusivity (MD) and fractional anisotropy (FA) maps were calculated using FMRIB’s Diffusion Toolbox^19^ and corticospinal tract masks were applied to the whole brain maps to calculate MD and FA within the corticospinal tract. Damage to the corticospinal tract was assessed using diffusion metrics (MD and FA) in the corticospinal tract on the side of stroke divided by diffusion metrics on the unaffected side (known as “MD and FA ratios”)^13^.

### Statistical analyses

The primary outcome measure was change in FMA-UE score between baseline and day-1 post therapy visit. All statistical analyses were performed using the Statistical Analysis System (SAS) version 9.4 (© 2002–2012 SAS Institute Inc.). We used the “PROC TTEST” function to assess differences in continuous baseline variables and FMA-UE change scores between treatment groups. We used Fisher’s exact test to assess differences in proportional variables, e.g., sex, hemisphere of stroke, between groups.

We used the “PROC GENMOD” function in SAS to perform general linear regression with standardized beta to identify predictors of post-treatment FMA-UE score i.e., a regression-based change analysis. Prediction variables of post-treatment FMA-UE score were: time since stroke, cortical versus subcortical stroke, months of rehabilitation pre-randomisation, WMH volume, cortical grey matter volume, cerebral normal appearing white matter volume, supratentorial cerebrospinal fluid (CSF) volume, MD and FA in the corticospinal tract on the side of stroke versus non-affected side (referred to as MD and FA ratios henceforth)^13^, stroke infarct volume, systolic and diastolic blood pressure. We performed separate models for each predictor because there was a high risk of unstable beta coefficients with many predictors versus relatively limited sample sizes, i.e., by including all predictors in a single model there would have been a large number of variables relative to sample size; this can lead to unreliable results via lower degrees of freedom. We adjusted all analyses by baseline FMA-UE score, sex, and age, and performed each analysis separately in the control and VNS treated groups. We did not perform multiple comparisons testing due to the exploratory nature of this study.

Finally, we defined “responders” as those who had greater than or equal to a 6-point improvement in FMA-UE score. Previous studies assessing FMA-UE using anchor-based methods estimated that the clinically important difference ranged from 4.24 to 7.25 points and a >50% improvement (excellent improvement) in overall arm and hand function has been shown to correspond to a FMA-UE change of 5.25 points^14^. We used *t*-tests to assess baseline FMA-UE scores and imaging characteristics in control responders versus VNS responders to assess whether characteristics of people who responded to VNS are different to those responding to traditional therapy techniques.

## Results

### Patient characteristics

**Figure 1:**
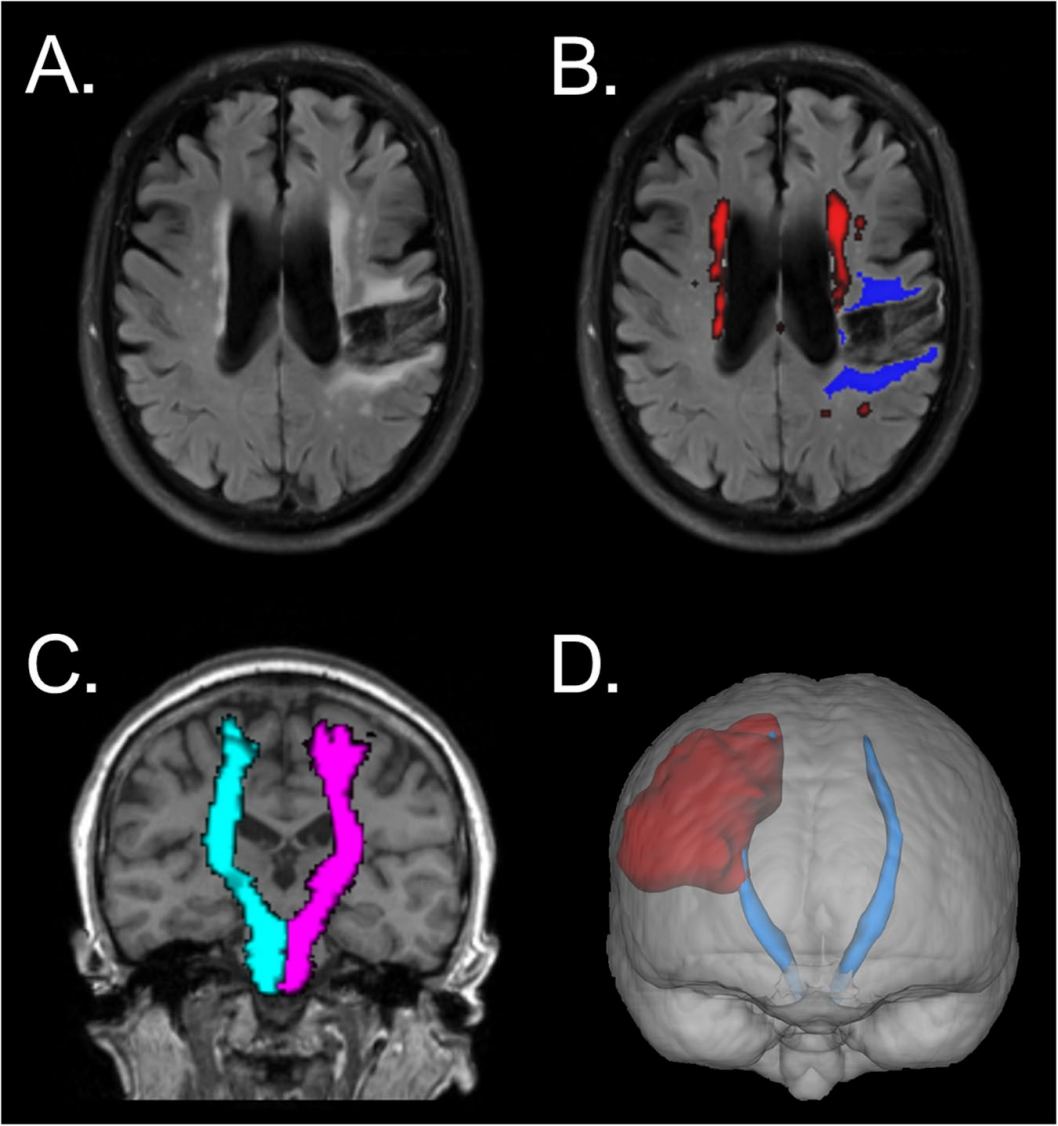
Illustration of brain MRI measures obtained. (**A**) Raw FLAIR image. (**B**) Segmentation of white matter hyperintensities (WMH; bright red) and ischaemic stroke infarct volumes (royal blue). (**C**) Estimate of the left (cyan) and right (pink) corticospinal tracts. (**D**) Interaction between stroke lesion (dull red) and corticospinal tract (light blue) in a 3D rendering. Images A and B come from the same patient, C and D are from separate patients and all are shown in neurological (“left is left”) convention.

Of the 37 randomised participants, 35 had brain MRI performed and were included in this analysis. There were no statistically significant differences in baseline characteristics between VNS-treated and control participants, except for cortical stroke incidence, which was higher in VNS participants (Table 1). WMH, stroke, and corticospinal tract image analysis results are illustrated in Fig. 1.

**Table 1:** Baseline patient characteristics.

Parameter	Rehabilitation only (N = 19)	VNS + Rehabilitation (N = 16)	Cohen’s *d*	*P*-value^a^
Age	59.6 ± 11.6	57.6 ± 12.5	0.1696	*P* = 0.6206
Male sex	N = 13	N = 10	N/A	*P* = 0.7362
Years since stroke	1.7 ± 1.2	1.6 ± 0.8	0.0721	*P* = 0.8331
Right dominant hand	N = 16	N = 15	N/A	*P* = 1.0000
Months of prior rehabilitation	20.5 ± 16.0	11.6 ± 13.5	0.5918	*P* = 0.1391
Right paretic limb	N = 9	N = 4	N/A	*P* = 0.2928
Cortical stroke	N = 6	N = 11	N/A	*P* = 0.0366*
Stroke on right hemisphere	N = 9	N = 11	N/A	*P* = 0.2844
Stroke on paretic side	N = 0	N = 0	N/A	*P* = 1.0000
Stroke on dominant side	N = 10	N = 12	N/A	*P* = 0.2654
Systolic BP mmHg	126.7 ± 9.9	126.4 ± 13.4	0.0276	*P* = 0.9356
Diastolic BP mmHg	79.5 ± 7.0	77.8 ± 10.8	0.1876	*P* = 0.5840
WMH volume ml	2.6 ± 3.2	3.6 ± 4.2	−0.2910	*P* = 0.4115
Stroke lesion volume ml	13.8 ± 20.4	20.7 ± 21.2	−0.3339	*P* = 0.3469
Cortical grey matter volume ml	434.2 ± 45.0	407.1 ± 50.2	0.5716	*P* = 0.1121
Cerebral white matter volume ml	426.8 ± 54.5	403.2 ± 74.0	0.3697	*P* = 0.2984
Supratentorial cerebrospinal fluid volume ml	287.5 ± 46.7	294.7 ± 33.0	−0.1758	*P* = 0.6185
CST MD affected side^b^	0.0010 ± 0.0001	0.0011 ± 0.0001	−0.4359	*P* = 0.2280
CST MD unaffected side	0.0009 ± 0.0001	0.0010 ± 0.0001	−0.1584	*P* = 0.6580
CST MD ratio^c^	1.09 ± 0.08	1.13 ± 0.07	−0.4919	*P* = 0.1752
CST FA affected side^b^	0.29 ± 0.03	0.27 ± 0.03	0.6798	*P* = 0.0645
CST FA unaffected side	0.32 ± 0.03	0.31 ± 0.02	0.4554	*P* = 0.2084
CST FA ratio^c^	0.89 ± 0.06	0.86 ± 0.06	0.5346	*P* = 0.1418
FMA-UE score	41.5 ± 9.5	35.1 ± 10.4	0.6434	*P* = 0.0667

Note: ^a^*P*-value is for difference between groups; ^b^mean diffusivity (MD) and fractional anisotropy (FA) in the corticospinal tract (CST) on the side of stroke; ^c^MD and FA in the corticospinal tract on the side of stroke versus non-affected side; *P < 0.05; BP = blood pressure; N/A = not applicable as Fisher’s exact test used for difference in proportions. WMH = white matter hyperintensity; FMA-UE = Fugl-Meyer Assessment, upper-extremity.

### Change in Fugl-Meyer Assessment, Upper-Extremity score between groups

VNS-treated participants had greater improvement in FMA-UE score at day-1 post therapy (8.63 ± 5.02 points) versus control (3.79 ± 5.04 points; t = 2.83, Cohen’s *d* = 0.96, *P* = 0.008). Baseline adjusted regression analysis also showed that improvement was greater in VNS participants (Beta = 4.98 points, standard error = 1.74, *P* = 0.0043).

### Predictors of follow-up FMA-UE score in control and VNS groups

**Figure 2:**
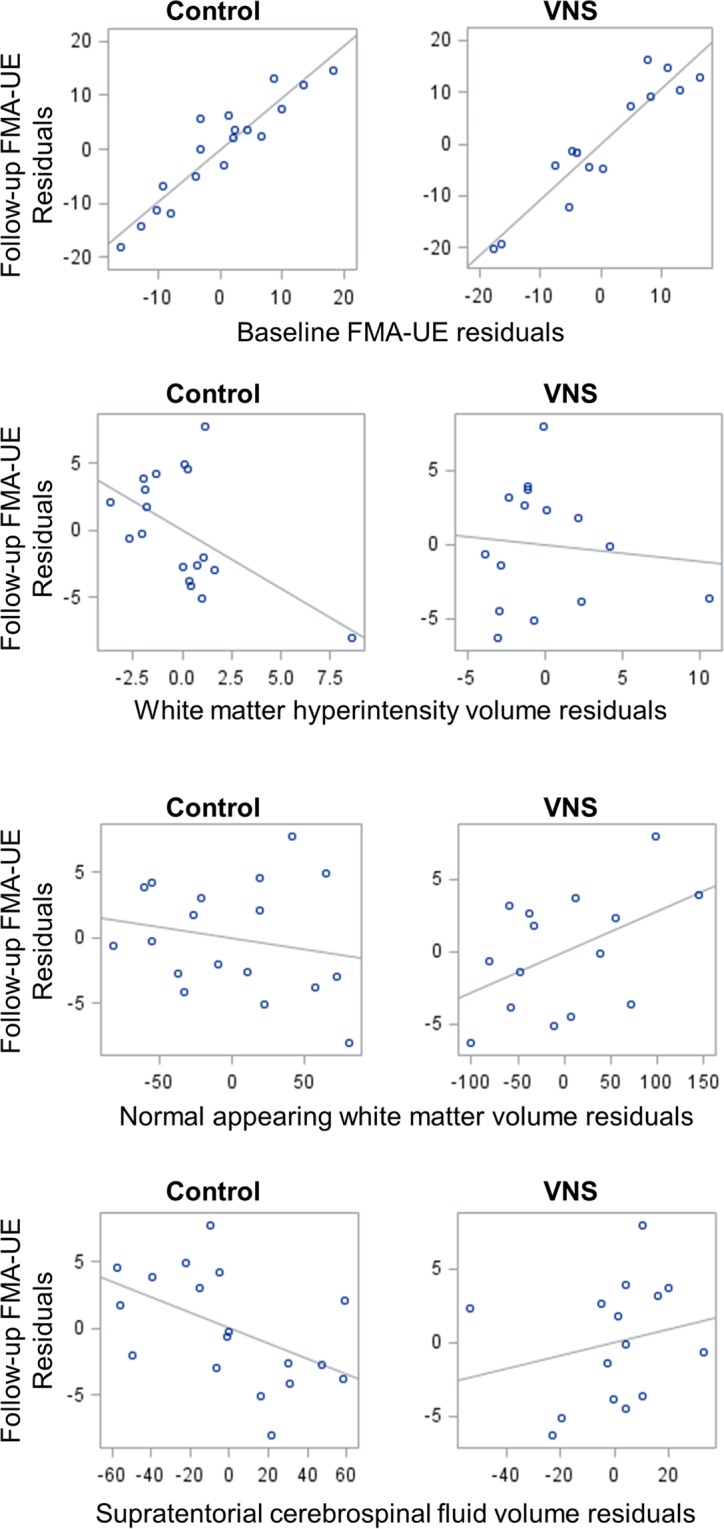
Partial regression plots showing adjusted associations with follow-up Fugl-Meyer Assessment, upper-extremity score (FMA-UE). Associations shown are between: 1. baseline FMA-UE and follow-up FMA-UE, adjusted for age and sex (top panel); 2. baseline white matter hyperintensity (WMH) volume and follow-up FMA-UE, adjusted for age, sex, and baseline FMA-UE (second top panel); 3. baseline normal appearing white matter volume and follow-up FMA-UE, adjusted for age, sex, and baseline FMA-UE (second bottom panel); 4. baseline supratentorial cerebrospinal fluid volume and follow-up FMA-UE, adjusted for age, sex, and baseline FMA-UE (bottom panel). Scatter plots are shown within the control (left panel) and vagus nerve stimulation (VNS; right panel) groups. Residuals for follow-up FMA-UE scores are calculated from the actual versus predicted follow-up FMA-UE scores in each model excluding the regressor shown on the x-axis, e.g., follow-up FMA-UE was predicted from age, sex, and baseline FMA-UE score in the second top, second bottom, and bottom panels. Residuals for brain imaging and baseline FMA-UE score are calculated from actual values minus predictions from remaining regressors in each model, e.g., for baseline WMH volume the residuals are actual WMH volume minus WMH volume predicted by age, sex, and baseline FMA-UE.

Predictors of follow-up FMA-UE score in control and VNS groups are shown in Table 2. Baseline FMA-UE score was the strongest predictor of six-week follow-up FMA-UE score in both groups: higher baseline scores were associated with higher follow-up scores. Higher pre-therapy WMH and CSF volumes were associated with less improvement in FMA-UE score in the control but not VNS group. Higher normal appearing white matter volume and lower systolic blood pressure were associated with greater improvement in FMA-UE score in the VNS but not control group. Adjusted associations between FMA-UE score and brain imaging predictors are illustrated in Fig. 2.

**Table 2:** Predictors of 6-week Fugl-Meyer Assessment, upper-extremity score (response) to control and VNS treatment.

Predictor	Control	VNS
Age	Beta = −0.07 ± 0.11 (*P* = 0.554)	Beta = −0.11 ± 0.09 (*P* = 0.223)
Male sex	Beta = −0.07 ± 0.23 (*P* = 0.757)	Beta = −0.23 ± 0.18 (*P* = 0.198)
Baseline Fugl-Meyer	**Beta = 0.87 ± 0.11 (** ***P*** < 0.0001)*	**Beta = 0.94 ± 0.09 (** ***P*** < 0.0001)*
Time since stroke	Beta = −0.01 ± 0.12 (*P* = 0.962)	Beta = −0.15 ± 0.09 (*P* = 0.118)
Cortical versus subcortical stroke	Beta = −0.14 ± 0.22 (*P* = 0.543)	Beta = −0.15 ± 0.22 (*P* = 0.488)
Months of rehabilitation pre-randomisation	Beta = −0.15 ± 0.15 (*P* = 0.315)	Beta = −0.01 ± 0.13 (*P* = 0.962)
White matter hyperintensity volume	**Beta = **−0.27 ± 0.10 (*P* = 0.006)*	Beta = −0.04 ± 0.10 (*P* = 0.690)
Cortical grey matter volume	Beta = −0.15 ± 0.11 (*P* = 0.159)	Beta = 0.142 ± 0.08 (*P* = 0.062)
Cerebral normal appearing white matter volume	Beta = −0.09 ± 0.10 (*P* = 0.389)	**Beta = 0.165 ± 0.08 (** ***P*** = 0.036)*
Supratentorial cerebrospinal fluid volume	**Beta = **−0.26 ± 0.11 (*P* = 0.013)*	Beta = 0.12 ± 0.13 (*P* = 0.373)
MD ratio^+^	Beta = −0.16 ± 0.10 (*P* = 0.101)	Beta = −0.01 ± 0.11 (*P* = 0.924)
FA ratio^+^	Beta = 0.10 ± 0.11 (*P* = 0.387)	Beta = −0.03 ± 0.10 (*P* = 0.786)
Stroke infarct volume	Beta = −0.07 ± 0.12 (*P* = 0.591)	Beta = −0.02 ± 0.11 (*P* = 0.874)
Systolic blood pressure	Beta = 0.03 ± 0.13 (*P* = 0.826)	**Beta = **−0.20 ± 0.03 (*P* = 0.033)*
Diastolic blood pressure	Beta = −0.18 ± 0.13 (*P* = 0.169)	Beta = 0.02 ± 0.12 (*P* = 0.851)

Note: *P < 0.05; Standard error of beta are shown after ± sign beside beta coefficients; ^+^mean diffusivity (MD) and fractional anisotropy (FA) in the corticospinal tract on the side of stroke versus non-affected side.

The association between WMH volume and FMA-UE score in the control group may have been driven by the outlying participant shown in the left panel of the second row in Fig. 2; the association was no longer statistically significant after removing this participant.

### VNS and control responders

There were 12/16 (75.00%) responders (increase of ≥6-points in FMA-UE) in the VNS group versus 6/19 (31.58%) responders in the control group (Fisher’s exact test *P* = 0.0176). The mean baseline FMA-UE score in VNS responders was 34.75 ± 9.64 points. The mean baseline FMA-UE score was 40.83 ± 7.63 points in control responders. The difference in baseline FMA-UE score between VNS and control responders was 6.08 points (*t* = 1.34, *P* = 0.198,). Responders in the VNS group had descriptively higher stroke (VNS = 21.12 ± 21.67 ml, control = 8.68 ± 18.40 ml, *P* = 0.2469), WMH (VNS = 2.94 ± 2.33 ml, control = 1.67 ± 0.80 ml, *P* = 0.1112), and CSF (VNS = 289.80 ± 33.59 ml, control = 256.20 ± 38.03 ml, *P* = 0.0727) volumes, and descriptively lower cortical grey matter (VNS = 404.30 ± 52.29 ml, control = 434.40 ± 45.98 ml, *P* = 0.2495) and normal appearing white matter (VNS = 402.10 ± 77.46 ml, control = 424.80 ± 56.59 ml, *P* = 0.5361) volumes at baseline.

## Discussion

In this combined study of data from two clinical trials, we found that VNS paired with rehabilitation was associated with greater improvement in FMA-UE score compared to rehabilitation alone. In a series of exploratory analyses, we found that, in controls, features of worse underlying brain health such as higher WMH volume and higher CSF volume were associated with worse outcome but this was not the case in people treated with VNS. Baseline FMA-UE score was lower and imaging findings less favourable in people who responded to VNS than in people who responded to physiotherapy alone. Although these differences were not statistically significant, several measures of brain health were consistently worse in VNS participants. This raises the possibility that VNS can provide additional benefit versus rehabilitation alone to people with greater baseline disability and cerebrovascular disease. These hypotheses need tested in prospective clinical studies.

We found that increased whole brain WMH and CSF volumes were associated with less improvement in FMA-UE score in control participants. This was not apparent in VNS treated participants and negative standard beta were several times larger in the control group versus the VNS group. One possible inference from these differential effects is that VNS can improve outcomes in people with cerebrovascular disease. VNS activates neurons in the basal forebrain and locus coeruleus and the ensuing reorganisation of cortical networks (via the release of acetylcholine and norepinephrine) may allow motor signals to bypass areas of white matter damage or tissue atrophy^5^. Consistent with this finding, we found higher normal appearing white matter volume in VNS participants was associated with higher improvement in FMA-UE. Higher systolic blood pressure was associated with less improvement in the VNS group but not the control group. VNS has previously been shown to reduce blood pressure^20^ but the reasons for this finding are unclear. These analyses were hypothesis generating and findings require confirmation in larger clinical trials. A phase III trial of VNS paired with upper-limb rehabilitation after stroke is underway.

The hypothesis that VNS may improve outcomes in those less likely to respond to conventional treatment, e.g., those with greater baseline disability, is supported by the fact that baseline FMA-UE was lower in VNS responders versus control responders. Although this difference (6.08 points) was not statistically significant, it is approximately equal to a level of clinical importance^6,7,14^. This likely reflects study power and the small sample size in this analysis (12 responders in VNS versus 6 in control). Additionally, VNS responders had descriptively worse structural brain health at baseline. Again, these differences were not statistically significant but each individual structural brain measure (including stroke, WMH, CSF and normal appearing tissue) was worse at baseline in VNS responders.

Our study has limitations principally related to the small sample of participants. Although the overall sample size was N = 35, we performed analyses within groups of N = 19 and N = 16. These sample sizes are clearly not large enough to draw robust conclusions. Limited sample sizes may produce spurious results or miss potentially true effects and that may be the case with the results we have presented. Indeed, the statistically significant association between WMH volume and FMA-UE score in the control group was no longer significant after removal of an outlying participant. Replication of this analysis in a larger sample is required to determine whether this association was no longer statistically significant due to decreased power or the outlying participant. Additionally, the developmental, rather than confirmatory, nature of this study meant we performed many statistical tests without multiple comparisons testing. Furthermore, it was difficult to determine whether variables were distributed statistically normal due to the limited number of participants. This further emphasises the need for caution in interpreting the results obtained. The VNS group had a larger proportion of participants with cortical stroke than the control group. Given we postulate that paired VNS causes task specific plasticity, the effect may differ in different stroke subtypes and between group differences could confound results. We have not seen a difference in response dependent solely on stroke subtype but the sample size to date does not allow this to be properly assessed. More work in a larger sample is required to determine whether cortical versus sub-cortical stroke participants are more likely to respond to VNS.

This study was not a hypothesis testing exercise, but an exploratory study designed to formulate hypotheses and present the totality of the only currently available data on VNS treatment for upper-limb weakness after stroke. These findings now need assessed in a large clinical trial.

While acknowledging the aforementioned limitations, our exploratory analyses have formed the hypotheses that VNS may provide additional benefit to patients with more severe baseline upper-limb disability and unfavourable imaging findings, such as higher CSF volume, that reduce the likelihood of success from therapy alone. A phase III trial that is currently underway will formally assess these hypotheses and the efficacy of VNS paired with rehabilitation for arm weakness after ischemic stroke.

## Data Availability

Data used in this analysis will remain confidential until one year after the commercial sponsor, MicroTransponder, Inc., obtains FDA approval or until regulatory approval has been abandoned.
